# Integrative genomics identifies new genes associated with severe COPD and emphysema

**DOI:** 10.1186/s12931-018-0744-9

**Published:** 2018-03-22

**Authors:** Phuwanat Sakornsakolpat, Jarrett D. Morrow, Peter J. Castaldi, Craig P. Hersh, Yohan Bossé, Edwin K. Silverman, Ani Manichaikul, Michael H. Cho

**Affiliations:** 1 Channing Division of Network Medicine, Department of Medicine, Brigham and Women’s Hospital, 181 Longwood Avenue, Room 451, Boston, MA 02115 USA; 2 Department of Medicine, Faculty of Medicine Siriraj Hospital, Mahidol University, Bangkok, Thailand; 3 Division of General Medicine, Department of Medicine, Brigham and Women’s Hospital, 181 Longwood Avenue, Room 451, Boston, MA 02115 USA; 4 Division of Pulmonary and Critical Care Medicine, Department of Medicine, Brigham and Women’s Hospital, 181 Longwood Avenue, Room 451, Boston, MA 02115 USA; 5 Department of Molecular Medicine, Institut universitaire de cardiologie et de pneumologie de Québec, Laval University, Quebec, Canada; 6 Department of Public Health Sciences, Center for Public Health Genomics and Biostatistics Section, University of Virginia, Charlottesville, VA USA

**Keywords:** Chronic obstructive pulmonary disease, Emphysema, Genome-wide association studies, Gene expression

## Abstract

**Background:**

Genome-wide association studies have identified several genetic risk loci for severe chronic obstructive pulmonary disease (COPD) and emphysema. However, these studies do not fully explain disease heritability and in most cases, fail to implicate specific genes. Integrative methods that combine gene expression data with GWAS can provide more power in discovering disease-associated genes and give mechanistic insight into regulated genes.

**Methods:**

We applied a recently described method that imputes gene expression using reference transcriptome data to genome-wide association studies for two phenotypes (severe COPD and quantitative emphysema) and blood and lung tissue gene expression datasets. We further tested the potential causality of individual genes using multi-variant colocalization.

**Results:**

We identified seven genes significantly associated with severe COPD, and five genes significantly associated with quantitative emphysema in whole blood or lung. We validated results in independent transcriptome databases and confirmed colocalization signals for *PSMA4*, *EGLN2*, *WNT3*, *DCBLD1*, and *LILRA3*. Three of these genes were not located within previously reported GWAS loci for either phenotype. We also identified genetically driven pathways, including those related to immune regulation.

**Conclusions:**

An integrative analysis of GWAS and gene expression identified novel associations with severe COPD and quantitative emphysema, and also suggested disease-associated genes in known COPD susceptibility loci.

**Trial registration:**

NCT00608764, Registry: ClinicalTrials.gov, Date of Enrollment of First Participant: November 2007, Date Registered: January 28, 2008 (retrospectively registered); NCT00292552, Registry: ClinicalTrials.gov, Date of Enrollment of First Participant: December 2005, Date Registered: February 14, 2006 (retrospectively registered).

**Electronic supplementary material:**

The online version of this article (10.1186/s12931-018-0744-9) contains supplementary material, which is available to authorized users.

## Background

Chronic obstructive pulmonary disease (COPD) is characterized by irreversible airflow obstruction and is strongly influenced by genetic factors [1, 2]. Genome-wide association studies of COPD and related traits (e.g., emphysema) have revealed multiple genetic loci associated with disease risk [3–5]. Most loci identified by genome-wide association studies (GWAS) are regulatory, and do not directly alter the amino acid sequence.

Gene expression is arguably the most impactful and well-studied effect of regulatory genetic variation. GWAS loci are enriched for expression quantitative trait loci (eQTL), rendering it a potential link between genetic variant and biology of disease [6, 7]. The efforts of large cohort studies and consortia such as the Genotype-Tissue Expression Project have discovered thousands of genetic variants associated with gene expression in multiple tissues. While most GWAS studies do not concomitantly measure gene expression, the strong relationship of genetic variation to gene expression allows one to use gene expression reference datasets to predict gene expression given a set of genotypes, and subsequently identify gene expression differences for a given phenotype. This approach has been implemented in software called S-PrediXcan and TWAS [8–10]. Aggregating information from variant level to infer gene-level associations increases the power to discover more genes at loci not previously implicated by GWAS and gives mechanistic insight regarding genes being regulated via disease-associated genetic variants [7, 11].

Despite the convention of naming a discovered locus for the nearest gene (e.g., *HHIP*), further study is needed to identify the specific gene(s) and variant(s) responsible for disease risk [9, 11, 12]. In identified COPD susceptibility loci, most loci contain multiple genes, and variants in these genes are correlated (in linkage disequilibrium). More than one gene in a locus may also play a role in disease pathogenesis, as seen in other complex diseases [13, 14]. With recently developed methods and a growing amount of gene expression data made publicly available, integrating GWAS with known functional annotations of each variant (e.g., associated with gene expression) could highlight novel and biologically relevant genes for further evaluation.

We hypothesized that application of these integrative methods to specific phenotypes of COPD (severe disease and quantitative emphysema) would facilitate discovery of new gene-disease associations and elucidate the mechanism of gene in existing susceptibility loci. Specifically, we sought to identify genes and pathways genetically up- or down-regulated by phenotype-associated variants in tissue-specific reference datasets using S-PrediXcan and TWAS [3, 5], and to assess the potential causality of individual genes using multi-variant colocalization.^1^

## Methods

### Genome-wide association studies and meta-analysis

**Fig. 1:**
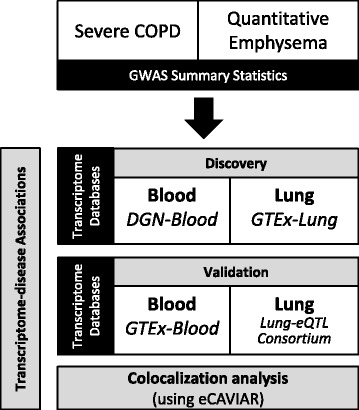
Summary of analyses. First, we discovered transcriptome-disease associations (predicted gene expression-disease) using reference data from DGN-Blood and GTEx-Lung. Then, we validated these associations using another set of reference data (GTEx-Blood and Lung-eQTL Consortium). Finally, we confirmed the transcriptome-disease associations using colocalization analysis. COPD = chronic obstructive pulmonary disease; DGN = Depression Gene Network; GTEx = Genotype-Tissue Expression project; Perc15 HU at 15th percentile of the density histogram; severe COPD is defined as FEV_1_ < 50% predicted and FEV_1_/FVC < 0.7

We used genome-wide association summary statistics for two phenotypes based on the same four cohorts. Demographic characteristics of individuals included in analyses of these two phenotypes are summarized in Tables 1 and 2. The four cohorts included individuals enrolled in Genetic Epidemiology of COPD (COPDGene, NCT00608764), Evaluation of COPD Longitudinally to Identify Predictive Surrogate Endpoints (ECLIPSE, SCO104960, NCT00292552), National Emphysema Treatment Trial (NETT) and Normative Aging Study (NAS), and GenKOLS (Genetics of COPD, Norway). Meta-analyses of these two phenotypes were published previously [3, 5]. Severe COPD was defined by post-spirometric measures of forced expiratory volume in 1st second (FEV_1_) lower than 50% of predicted value and the ratio of FEV_1_ to forced vital capacity (FEV_1_/FVC) less than 0.7, excluding individuals with known severe alpha-1 antitrypsin deficiency. For quantitative emphysema, we produced the histogram of segmented CT chest images and used the percentage low attenuation area at − 950 Hounsfield units (HU) threshold (%LAA-950), and the HU at the 15th percentile of the density histogram (Perc15) for the quantification of emphysema. A summary of our approach is shown in Fig. 1.

**Table 1:** Demographic characteristics of individuals in the analysis of severe COPD

	COPDGene-NHW		COPDGene-AA		ECLIPSE		NETT/NAS		Norway GenKOLS	
	Cases	Control Subjects	Cases	Control Subjects	Cases	Control Subjects	Cases	Control Subjects	Cases	Control Subjects
N	1390	2534	352	1749	999	178	373	435	383	808
Age, years (SD)	65.2 (7.8)	59.5 (8.7)	60.6 (8.1)	52.8 (6.0)	63.5 (7.0)	57.5 (9.4)	67.5 (5.8)	69.8 (7.5)	66.7 (9.7)	55.6 (9.7)
Smoking pack-years (SD)	58.7 (28.4)	37.8 (20.3)	43.9 (23.4)	36.4 (20.1)	50.7 (26.3)	32.1 (24.8)	66.4 (30.7)	40.7 (27.9)	33.0 (19.9)	19.7 (13.6)
FEV_1_, % predicted (SD)	34.0 (9.9)	96.8 (11.0)	34.8 (10.4)	98.4 (12.2)	36.5 (8.6)	107.8 (13.6)	28.1 (7.4)	100.0 (13.2)	34.4 (10.3)	94.9 (9.2)
FEV_1_/FVC (SD)	0.390 (0.103)	0.779 (0.050)	0.430 (0.105)	0.800 (0.052)	0.387 (0.095)	0.790 (0.053)	0.324 (0.064)	0.793 (0.0527)	0.412 (0.108)	0.791 (0.043)
Men, n (%)	803 (58)	1250 (49)	204 (58)	1017 (58)	698 (70)	103 (58)	238 (64.0)	435 (100)	235 (61)	405 (50)

*Definition of abbreviations*: *COPD* chronic obstructive pulmonary disease, *COPDGene* Genetic Epidemiology of COPD, *ECLIPSE* Evaluation of COPD Longitudinally to Identify Predictive Surrogate Endpoints, *GenKOLS* Genetics of COPD, Norway, *NETT* National Emphysema Treatment Trial, *NAS* Normative Aging Study

**Table 2:** Demographic characteristics of individuals in the analysis of quantitative emphysema

	COPDGene-NHW		COPDGene-AA		ECLIPSE		NETT	Norway GenKOLS	
	Cases	Noncases	Cases	Noncases	Cases	Control Subjects	Cases	Cases	Control Subjects
N	3243	3062	901	2132	1393	145	332	417	406
Age, years (SD)	64.4 (8.3)	59.7 (8.6)	58.6 (8.1)	53 (6.0)	63.4 (7.0)	57.3 (9.4)	67.4 (5.9)	64.2 (9.3)	55.6 (9.4)
Smoking pack-years (SD)	54.4 (27.5)	39.7 (21.5)	42 (23.1)	36.6 (20.5)	49.8 (26.7)	31.8 (26.6)	65.8 (30.8)	31 (18.2)	19.8 (14.1)
Men, n (%)	1832 (56.5)	1462 (47.7)	497 (55.2)	1209 (56.7)	911 (65.4)	85 (58.6)	212 (63.9)	263 (63.1)	216 (53.2)
Current Smokers, n (%)	1199 (37)	1263 (41.2)	595 (66)	1838 (86.2)	480 (34.5)	58 (40.0)	0 (0)	210 (50.4)	164 (40.4)
FEV_1_, % predicted (SD)	57.4 (23.0)	91.3 (14.8)	59.5 (22)	92.2 (16.5)	47.4 (15.5)	108.6 (13.4)	28.2 (7.3)	52.5 (16.9)	94.9 (9.2)
FEV_1_/FVC (SD)	0.517 (0.136)	0.779 (0.050)	0.550 (0.120)	0.800 (0.052)	0.442 (0.115)	0.792 (0.055)	0.317 (0.061)	0.523 (0.125)	0.791 (0.042)
LAA-950, % (range)	7.5 (0–61.9)	1.2 (0–26.9)	4.6 (0–61.2)	0.7 (0–35.8)	16.3 (0.1–58.7)	2.3 (0.1–14.2)	15 (0.3–49.9)	7 (0–53.2)	0.5 (0–34.4)
Perc15, HU (SD)	− 938.1 (26.8)	−909.9 (22.8)	− 926.5 (32)	− 893.4 (28.1)	−950.9 (25.9)	− 906.2 (25.9)	− 949.7 (17.8)	− 932.8 (30.2)	−891.6 (26.3)

Cases are GOLD grade 1 or more severe (as in NETT) cases; control subjects are smokers who have normal spirometry (GOLD grade 0); noncases include GOLD grade 0 and PRISm (preserved ratio, impaired spirometry) subjects

*Definition of abbreviations*: *LAA-950* low attenuation area using a threshold of − 950 HU, *COPD* chronic obstructive pulmonary disease, *COPDGene* Genetic Epidemiology of COPD, *ECLIPSE* Evaluation of COPD Longitudinally to Identify Predictive Surrogate Endpoints, *GenKOLS* Genetics of COPD, Norway, *GOLD* Global Initiative for Chronic Obstructive Lung Disease, *HU* Hounsfield units, *NETT* National Emphysema Treatment Trial, *Perc15* HU at the 15th percentile of the density histogram

### Integration of GWAS and gene expression

To integrate our GWAS and gene expression results, we used S-PrediXcan [10]. We included two relevant reference transcriptome databases in our analysis, whole blood from Depression Genes and Networks (DGN-Blood) and lung tissue from Genotype-Tissue Expression consortium (GTEx-Lung). Details on prediction models and datasets used were provided in Additional file 1: Supplementary Methods. The ability of genetic variants to predict the expression of individual genes varies; only genes with significant prediction models were included in the analysis (11,529 genes for DGN-Blood and 6425 genes for GTEx-Lung). We accounted for multiple hypothesis testing using Bonferroni correction to determine statistical significance of gene-disease associations, resulting in *p*-value of 4.34 × 10^− 6^ and 7.78 × 10^− 6^ for DGN-Blood and GTEx-Lung, respectively.

### Validation in other reference transcriptome databases

To determine whether our imputed gene expression was consistent in other datasets, we tested significant genes from DGN-Blood and GTEx-Lung in two independent reference transcriptome databases, GTEx for whole blood (GTEx-Blood) and the Lung-eQTL Consortium for lung tissue using S-PrediXcan and TWAS/FUSION (Additional file 1: Supplementary Methods). We considered an expression result to be validated if the direction of effect was consistent and the Bonferroni-corrected *P*-value < 0.05.

### Colocalization analysis using eCAVIAR

Colocalization analysis estimates a posterior probability that a given variant or set of variants is causal for both the phenotype of interest (e.g., COPD) and expression level of a given gene. We used eCAVIAR (eQTL and GWAS Causal Variant Identification in Associated Regions), as it allows for multiple causal variants [15]. Details on parameters and procedures used in the analysis were present in Additional file 1: Supplementary Methods. Genes identified in whole blood were tested for colocalization using eQTL from GTEx-Blood while using GTEx-Lung and Lung-eQTL Consortium for lung tissue. The probability of a variant to be causal for a given gene in both datasets was determined by the colocalization posterior probability (CLPP) that approximates the posterior probability of a variant to be causal in GWAS and posterior probability of a variant to be causal in eQTL [15]. We also obtained functional annotations of colocalized variants in lung relevant cell types (Additional file 1: Supplementary Methods).

## Results

### Severe COPD

**Fig. 2:**
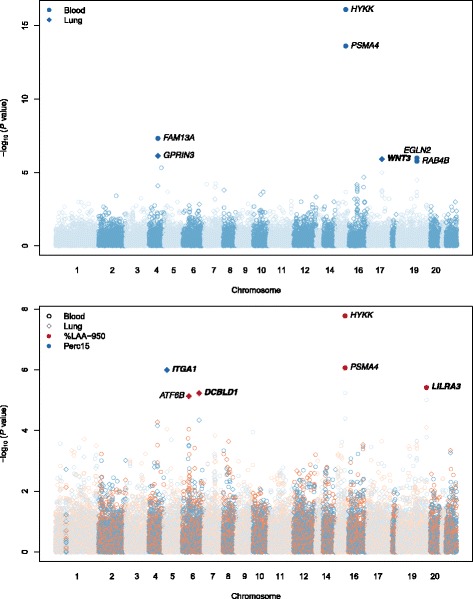
Manhattan plots of associations of imputed gene expression and phenotypes (severe COPD in the upper panel; %LAA-950 and Perc15 in the lower panel). Color indicates phenotypes and shape indicates tissue (see figure legend)

We first examined the association between severe COPD and imputed gene expression. Significant associations based on gene-based Bonferroni corrections for DGN-Blood and GTEx-Lung are shown in Table 3 and Fig. 2.

**Table 3:** Result of association analysis between imputed gene expression and severe COPD and emphysema (%LAA-950 and Perc15) with validation

Genetic Loci	Phenotype	Gene	Tissue	Discovery Z score	Discovery *P* value	Validation Z score	Validation *P* value
4q22	Severe COPD	*FAM13A*	Blood	−5.46	4.80E-08	NA	NA
4q22	Severe COPD	*GPRIN3*	Lung	4.95	7.40E-07	0.94	3.40E-01
5q11	Perc15	*ITGA1*	Lung	−4.89	1.00E-06	NA	NA
6p21	%LAA-950	*ATF6B*	Lung	−4.48	7.30E-06	−1.33	1.80E-01
6q22	%LAA-950	*DCBLD1*	Lung	4.53	5.80E-06	3.81	1.40E-04
15q25	Severe COPD	*HYKK*	Blood	−8.33	8.20E-17	NA	NA
15q25	Severe COPD	*PSMA4*	Blood	7.62	2.50E-14	7.57	3.80E-14
15q25	%LAA-950	*HYKK*	Blood	−5.64	1.70E-08	NA	NA
15q25	%LAA-950	*PSMA4*	Blood	4.92	8.50E-07	5.1	3.40E-07
17q21	Severe COPD	*WNT3*	Lung	4.85	1.20E-06	4.6	4.30E-06
19q13	Severe COPD	*EGLN2*	Blood	4.89	1.00E-06	4.35	1.30E-05
19q13	Severe COPD	*RAB4B*	Blood	−4.78	1.70E-06	−3.82	1.30E-04
19q13^a^	%LAA-950	*LILRA3*	Blood	−4.62	3.80E-06	−4.13	3.60E-05

Lung = Genotype Tissue Expression (GTEx) consortium lung, Blood = Depression Genes and Networks (DGN) blood, NA = prediction model not available in the validation dataset (see Discussion)

^a^The two loci at 19q13 are separated by 13 MB (see text)

**Fig. 3:**
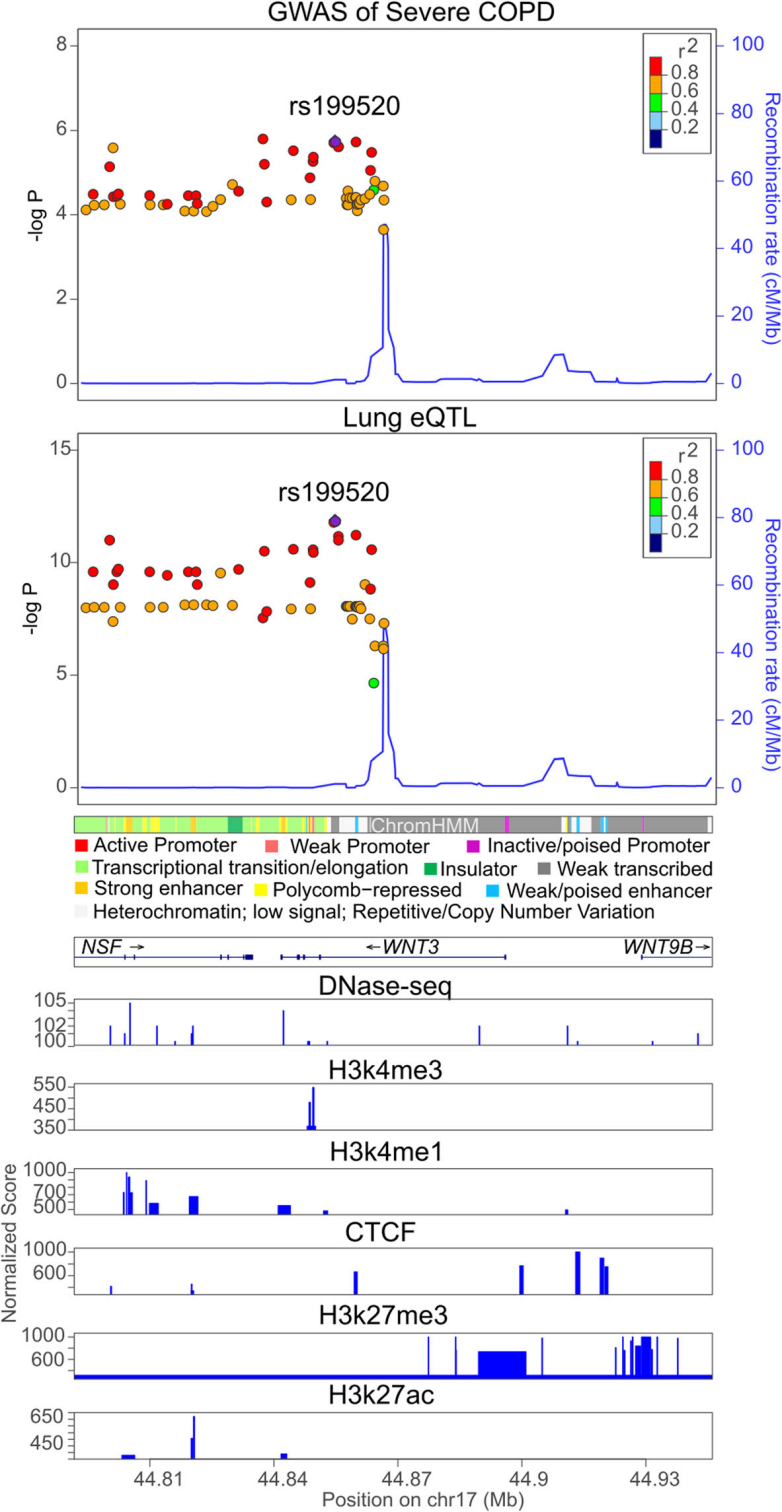
Regional association plots within 50 kb of *WNT3.* GWAS of severe COPD and lung eQTL are shown in the upper panel. Chromatin states and epigenomic marks of normal human lung fibroblasts are shown in the lower panel (see Additional file 1: Supplementary Methods)

In the whole blood reference dataset from DGN, we identified five significant genes: *FAM13A* in 4q22 (*P* = 4.81 × 10^− 8^), *HYKK* and *PSMA4* in 15q25 (*P* = 8.16 × 10^− 17^ and 2.47 × 10^− 14^, respectively), and *EGLN2* and *RAB4B* in the 19q13 locus (*P* = 1.03 × 10^− 6^ and 1.72 × 10^− 6^, respectively). All of these genes are located in COPD susceptibility loci previously reported in the literature [4, 16]. In lung tissue, we identified two genome-wide significant genes, *GPRIN3* in the 4q22 locus (*P* = 7.43 × 10^− 7^) and *WNT3* in the 17q21 locus (*P* = 1.24 × 10^− 6^); the latter locus was not identified in the single variant GWAS of severe COPD (Fig. 3).

### Emphysema

In whole blood and lung tissue, we identified five genes significantly associated with %LAA-950 and one gene with Perc15 (Table 3; Fig. 2). We found two significant associations of genes at loci previously associated with %LAA-950, *PSMA4* in 15q25 and *ATF6B* in 6p21, the latter which is located near *AGER.* The top genome-wide significant variant at this latter locus – which lies within the HLA (Human Leukocyte Antigen) region – is a nonsynonymous variant in *AGER;* however, *AGER* was not significant in either blood or lung (*P* = 0.81 and 0.18, respectively). *LILRA3*, *DCBLD1*, and *ITGA1* are at loci not previously associated with COPD or emphysema.

### Validation in other reference transcriptome databases

To provide further evidence for differentially expressed genes associated with severe COPD and emphysema, we repeated our analysis using additional reference transcriptome databases with the same GWAS data. In blood, we validated *PSMA4*, *EGLN2*, and *RAB4B* for severe COPD (*P* = 3.79 × 10^− 14^, 1.34 × 10^− 5^, and 1.33 × 10^− 4^, respectively), and *PSMA4* and *LILRA3* for %LAA-950 (*P* = 3.37 × 10^− 7^ and 3.62 × 10^− 5^, respectively) by using GTEx-Blood as a validation for genes identified through whole blood transcriptome analysis (Table 3). We also validated *WNT3* for severe COPD (*P* = 4.27 × 10^− 6^) and *DCBLD1* for %LAA-950 (*P* = 1.41 × 10^− 4^) for genes identified from GTEx-Lung using a lung transcriptome database from Lung-eQTL Consortium (Table 3). We also noted that for several genes, a prediction model was not available, likely due to lower power and sample size in the validation dataset for whole blood [9]. Although the association of *FAM13A* was initially identified using blood dataset, its association was significant using Lung-eQTL Consoritium (Z score = 4.52, *P* = 6.3 × 10^− 6^).

### Colocalization analysis of validated genes

Gene expression differences identified using S-PrediXcan may be causally associated with the phenotype of interest, but also can be due to linkage disequilibrium (LD) [15]. To determine whether there was evidence of shared causality, we performed colocalization analysis, using a method that allows for multiple causal variants. Of the seven associations, six occupied at least one shared variant (Table 4): *PSMA4*, *EGLN2*, and *WNT3* (Fig. 3) for severe COPD; *PSMA4*, *LILRA3* (Additional file 1: Figure S1), and *DCBLD1* (Additional file 1: Figure S2) for %LAA-950. For associations identified in lung, we additionally confirmed the colocalization signals using the Lung-eQTL consortium dataset (Additional file 1: Table S1). We then sought to leverage functional annotation of shared variants especially for those with high colocalization probability. Some colocalized variants associated with *PSMA4*, *LILRA3*, *DCBLD1*, and *WNT3* located in annotated regulatory regions (e.g., rs35061187 is in active transcription start site (TSS) in lung fibroblasts) or predicted to affect transcription factor binding (Additional file 1: Table S1 and S2).

**Table 4:** Colocalized variants in validated genes and association statistics in corresponding GWAS and eQTL datasets

Locus	Phenotype	Gene	Tissue	Top colocalized variant	Variant CLPP	GWAS *P* value	eQTL *P* value
6q22	%LAA-950	*DCBLD1*	Lung	rs34882116	0.13	2.87E-05	1.28E-11
15q25	Severe COPD	*PSMA4*	Blood	rs56077333	0.21	1.57E-18	4.16E-06
15q25	%LAA-950	*PSMA4*	Blood	rs56077333	0.14	2.64E-09	4.16E-06
17q21	Severe COPD	*WNT3*	Lung	rs199520	0.21	1.75E-06	1.30E-12
19q13	Severe COPD	*EGLN2*	Blood	rs35755165	0.12	9.91E-09	1.87E-06
19q13	%LAA-950	*LILRA3*	Blood	rs380267	0.28	3.32E-05	4.54E-47

CLPP = Colocalization posterior probability, the probability that a particular variant is causal in both GWAS and eQTL. A CLPP of 0.01 or greater previously demonstrated high accuracy and precision (see Additional file 1: Supplementary Methods), [15]. Only top colocalized variants, a variant with highest CLPP for each gene/tissue, are shown

### Genetically regulated differential expression of genes in known susceptibility loci

Of the above significantly differentially regulated genes, four are in known susceptibility loci (4q22 and 15q25 with severe COPD, and 6p21 and 15q25 with %LAA-950). We also sought to investigate whether additional known susceptibility loci for severe COPD and quantitative emphysema affect the genetically regulated expression of nearby genes. We investigated nominal association results (*P* < 0.05) in other nine susceptibility loci in either discovery or validation datasets. Using this criterion, we found 5 additional suggestive associations, namely *TGFB2* (1q41), *HHIP* (4q31)*,* and *RIN3* (14q32.12) with severe COPD, and *HHIP* (4q31) with %LAA-950 and Perc15 (Additional file 1: Table S3). However, we did not find any suggestive signals in 11q22 (*MMP12*) with severe COPD, 14q32.13 (*SERPINA10*) with %LAA-950, and 8p22 (*DLC1*) with %LAA-950 and Perc15.

### Pathway enrichment analysis

In contrast to genetic gene set enrichment methods that rely only on the location of the SNP to infer affected genes [17], we used the results of our predicted gene expression to identify pathways by using the top 1% of differentially expressed genes (Table 5, Additional file 1: Supplementary Methods). We identified enrichment of the T cell receptor signaling pathway (corrected *P* = 6.6 × 10^− 3^); this pathway included *PSMA4* along with genes in the HLA complex. We also found significant enrichment for proteasome core complex genes (corrected *P* = 2.82 × 10^− 2^) which included *PSMF1*, *PSMB4*, and *PSMB9*. An additional pathway of interest was cell-matrix adhesion of collagen binding (corrected *P* = 2.74 × 10^− 3^) (Table 5). We also found enrichment of the asthma pathway using the KEGG database (corrected *P* = 4.80 × 10^− 3^), containing *MS4A2* and genes in HLA.

**Table 5:** Selected results of pathway enrichment analysis based on predicted differential gene expression

Phenotype	Tissue	Functional category	*P* value for enrichment*	Overlapping genes
Severe COPD	Lung	collagen binding involved in cell-matrix adhesion	2.70E-03	*ITGA10*, *ITGA2*, *ITGA1*
Severe COPD	Lung	proteasome core complex	2.80E-02	*PSMF1*, *PSMB4*, *PSMB9*
Severe COPD	Lung	translation factor activity, RNA binding	3.70E-02	*TCEB3*, *EEFSEC*, *TUFM*, *EIF3C*, *EIF3CL*
%LAA-950	Whole Blood	MHC class II protein complex	5.20E-05	*HLA-DQB1*, *HLA-DRB1*, *HLA-DQA1*, *HLA-DQB2*, *HLA-DQA2*
%LAA-950	Whole Blood	PD-1 signaling	6.30E-04	*HLA-DQB1*, *HLA-DRB1*, *HLA-DQA1*, *HLA-DQB2*, *HLA-DQA2*
%LAA-950	Whole Blood	Downstream TCR signaling	8.00E-04	*PSMA4*, *NFKBIA*, *BCL10*, *HLA-DQB1*, *HLA-DRB1*, *HLA-DQA1*, *HLA-DQB2*, *HLA-DQA2*
%LAA-950	Whole Blood	Asthma	4.80E-03	*MS4A2*, *HLA-DQB1*, *HLA-DRB1*, *HLA-DQA1*, *HLA-DQA2*
%LAA-950	Whole Blood	T cell receptor signaling pathway	6.60E-03	*PSMA4*, *NFKBIA*, *BCL10*, *HLA-DRB1*, *HLA-DQA1*, *PVRIG*, *HLA-DQB2*, *HLA-DQA2*
Perc15	Lung	negative regulation of ERBB signaling pathway	4.50E-02	*NRG1*, *ITGA1*, *UBA52*

*P values were corrected for multiple testing using g:SCS method (see Additional file 1: Supplementary Methods)

## Discussion

Genome-wide association studies have arguably become the mainstay of identifying genetic risk factors for complex disease. However, these studies cannot identify which gene(s) in the region is responsible for the association, and testing all variants individually and independently is likely suboptimal. Here, we used an integrative method that combines the genetic component of gene expression with genetic association analysis in severe COPD and quantitative emphysema to predict differentially expressed genes. Importantly, this method focuses on the association of genetic component of gene expression, not gene expression as a whole, as is typical in most gene expression studies. We also provided additional support of our results by examining results in a second gene expression dataset, and performing colocalization analysis that attempts to identify whether association signals for gene expression and a phenotype of interest appear to be driven by the same causal variant(s). We implicated genes that are genetically regulated in known COPD-susceptibility loci, such as *FAM13A*, and also found genes in regions that were not previously reported: *WNT3* for severe COPD, and *DCBLD1* and *LILRA3* for quantitative emphysema.

We found a novel association of *WNT3* in lung tissue with severe COPD in two gene expression datasets. Although variants surrounding this gene in the 17q21 locus were not genome-wide significant in our COPD analysis GWAS (Fig. 3), the top signal (rs9912530) is in strong LD with variants previously reported in GWAS of FEV_1_ [18, 19], interstitial lung disease [20], and idiopathic pulmonary fibrosis [21] (r^2^ with these previously described variants, 0.55–0.72). *WNT3* (Wnt family member 3) encodes Wnt3, a critical component of the Wnt-beta-catenin-TCF signaling pathway [22] and a required signal for the apical ectodermal ridge in limb patterning [23]. Deficient *WNT3* is associated with tetra-amelia syndrome, a Mendelian disease characterized by an absence of all limbs. The top signal is also in strong LD with variants associated with various complex diseases such as Parkinson’s disease and celiac disease (r^2^ 0.72–0.79) [24, 25]. Previous expression studies of small airway epithelium found that this gene, along with its Wnt signaling companions, was down-regulated in smokers compared with nonsmokers [26]. Of interest, *FAM13A*, a well-supported COPD susceptibility gene, has been involved in the Beta-catenin/Wnt signaling pathway by protein degradation [27]. While there is substantial interest in Wnt signaling in lung disease [28], the contribution of *WNT3* to the pathogenesis of COPD requires further investigation. To address whether these findings were specific for severe COPD, we repeated the analysis including moderate disease (GOLD 2). All of our genes were at least nominally significant, though overall the significance of our findings was attenuated (Additional file 1: Table S4).

For emphysema, we identified novel associations of *LILRA3* and *DCBLD1* using whole blood and lung tissue, respectively, and validated these findings in additional gene expression datasets. *LILRA3* (leukocyte immunoglobulin like receptor A3) is a gene encoding a soluble receptor for class I major histocompatibility complex (MHC) antigens expressed in monocytes and B cells, which is located in the 19q13 locus. Our top hit from GWAS in this locus, was not genome-wide significant (rs384116 with *P* = 1.88 × 10^− 5^; Additional file 1: Figure S1), and 13-Mb away from the previously reported locus [16] that contains *EGLN2* and *RAB4B* (rs7937; r^2^ 0.002). It is in modest LD with variants suggestively associated with FEV_1_/FVC [18] (r^2^ 0.44), in strong LD with variants genome-wide significantly associated with HDL-C level [29] and prostate cancer [30] (r^2^ 0.92–0.99). Blood may be the most relevant tissue for this gene, as it is preferentially expressed [31] with a high estimate of heritability of gene expression in whole blood [32]. However, it may also have an effect in other tissues, given its broad eQTL effects identified by multi-tissue eQTL analysis [33]. This was supported by the suggestive signals of this gene using lung tissue in S-PrediXcan analysis (*P* = 7.71 × 10^− 5^ in GTEx-Lung and 1.38 × 10^− 4^ in the Lung-eQTL Consortium with the same direction of effect). Nonetheless, its functional role in COPD has not been described previously. Our other novel association identified in lung tissue, *DCBLD1* (discoidin, CUB and LCCL domain containing 1), located in the 6q22 locus, is an integral component of cell membranes and binds to oligosaccharides [34]. GWAS signals in this locus are also sub-genome wide significant (Additional file 1: Figure S2). Our top GWAS variant at this locus was in LD with variants associated with lung cancer [35] (r^2^ 0.54).

In addition to novel associations, our study also provides insight into disease-associated genes in known COPD susceptibility loci. We identified six genes (*FAM13A*, *GPRIN3*, *HYKK*, *PSMA4*, *EGLN2*, and *RAB4B*) in three known COPD-susceptibility loci for which their genetic component of gene expression in blood or in lung tissue is associated with severe COPD. Five of these six genes are not the most proximal to the top associated SNP, a phenomenon previously observed in other genetic association studies [36, 37]. These findings underscore the complexity of genetic regulation in tissues and also identify multiple potential effector genes in the same locus. For example, in 15q25, *PSMA4*, and not *CHRNA3* (the nearest gene to the top GWAS hit) was highlighted in S-PrediXcan and colocalization analysis. Although a role for *IREB2* has been clearly demonstrated [38], our study suggested that other genes in the locus, particularly *PSMA4* – a gene encoded for subunit of proteasome complex that acts in the proteolytic pathway [39], may also be of biologic importance.

At the 4q22 locus, an association for *FAM13A* identified using DGN-Blood was not validated in the GTEx-blood dataset. However, a significant but directionally opposite association was identified in the Lung-eQTL consortium dataset. To further explore this phenomenon, we examined individual SNP eQTL data from the Framingham Heart Study (FHS) blood, and the lung tissue from the Lung eQTL consortium (Additional file 1: Supplementary Methods). We confirmed that SNPs have opposite directions of effect in lung and blood (Additional file 1: Figure S3 and S4). This finding is consistent with prior reports describing significant and opposite tissue specific effects of eQTLs [33, 40, 41]. The interpretation of this phenomenon is not clear, but may be a result of pleiotropic effects of *FAM13A* [42, 43]. Of note, a recent analysis of emphysema-related gene expression in blood and lung tissue [44] found that the expression of genes in two tissues are often opposite; together, our findings highlight the tissue-specific genetic regulation of genes in COPD susceptibility loci. At the 19q13 locus, while both *EGLN2* and *RAB4B* were successfully validated, only GWAS and eQTL signals for *EGLN2* colocalized. This genetic locus was associated with COPD [16] and smoking behavior [45]. Although the causal gene(s) in this region is unclear, methylation and expression studies support the role of *EGLN2* in this region [46]. *EGLN2* (egl-9 family hypoxia inducible factor 2) encodes an enzyme that regulate the degradation of alpha subunit of hypoxia inducible factor (HIF) [47]. Gene and protein expression of HIF-1α is reduced in lung tissue samples from COPD patients [48].

Although *ATF6B* (activating transcription factor 6 beta) and *ITGA1* (integrin subunit alpha 1) were not successfully validated, we cannot rule out the possibility of false negatives due to differences between the transcriptome datasets used for validation, and they are potentially interesting candidates for COPD. *ATF6B* was implicated in the unfolded protein response (UPR) pathway during endoplasmic reticulum (ER) stress following cigarette smoke, and may contribute to lung inflammation in patients with COPD [49], while integrins were found to be involved in COPD through the mitogen-activated protein kinase (MAPK) pathway [50, 51]. This region also harbors variants associated with FEV_1_/FVC [52]. Decreased expression of *ITGA1* was observed in the small airways of patients with low FEV_1_ [53].

Our analysis assesses only the genetic component of gene expression. We also investigated whether these genes were differentially expressed in COPD patients, in 464 blood samples from the COPDGene study [54], and 151 lung tissue samples [55] (Additional file 1: Supplementary Methods and Table S5-S8). These genes were not differentially expressed, with the exception of *LILRA3*, which was nominally significant with %LAA-950 (*P* = 0.03). Given that the genetic component of gene expression was replicated, we believe that the genetic findings are robust, and speculate that these null findings could be due to non-genetic (i.e. environmental) perturbations that may occur downstream, or as a result of the genetic effects. In fact, in several cases measurements of mRNA or protein are actually opposite those predicted by genetic risk. For example, *SERPINA1* risk alleles result in decreased levels and increased risk for COPD, yet average, alpha-1 levels in patients with COPD are actually elevated. Similarly, genetic variants in *AGER* and *DSP* affect transcript or protein levels opposite than what is measured in disease [4, 56, 57]. The mechanisms underlying our genetic findings, as well as *AGER* and *DSP*, that result in null or opposite direction effects requires further experimental investigation.

In addition to examination of individual loci, we applied pathway enrichment analysis to nominally significant differentially expressed genes in severe COPD and quantitative emphysema both in whole blood and lung tissue. This analysis identified enrichment of the T cell receptor signaling pathway in emphysema. This finding is consistent with reports that found antigen-specific T cell differentiation in lungs of patients with severe emphysema [58]. Our analysis using gProfileR does not assess of direction of effect, and the relative up- or down-regulation of specific genes in this pathway makes determination of direction difficult. To attempt to infer direction, we used Gene Set Enrichment Analysis (GSEA; [59]). In these results, the TCR signaling pathway and downstream TCR response were up-regulated, though these results were not statistically significant (Additional file 1: Table S9). Further study will be needed to determine the combined effects of COPD genetic susceptibility variants on T cell function and whether these explain some immune dysfunction seen in COPD [60, 61]. The finding of the enrichment of genes in the proteasome core complex further suggested a role of proteasome in COPD as described previously. Somewhat surprisingly, we observed enrichment of the asthma pathway in KEGG using genes identified in quantitative emphysema. This finding complements the description of substantial genetic correlation of COPD and asthma [4], and the presence of quantitative emphysema (or lung hyperinflation) in asthmatic patients [62].

Our study did not identify associations of genetically regulated differential expression of genes at some previously reported GWAS loci. Moreover, some of our identified associations in our discovery dataset were not successfully validated in a second transcriptome dataset. These findings indicate some of the limitations of our approach. First, as S-PrediXcan uses *cis* genetic variants as predictors for gene expression, variants that have lesser or no effect on transcript abundance or act in *trans* would not be detected by this approach [63]. Second, although most genetic variants implicated by GWAS are likely regulatory, only a minority of genetic loci are explained by existing eQTLs [64]. This may be due to lack of data in the appropriate tissue, cell type, or biologic conditions; or the heterogeneity of gene expression studies of bulk tissue. We may overcome these issues as more gene expression datasets and newer techniques such as single-cell gene expression profiling [65] become widely available. Moreover, issues such as cell type composition, sample collection methods, disease status, and differences in analytic methods also made the overlapping analysis challenging. Third, the number of genes available for an analysis depends on the power and sample size of the expression data used in constructing a gene expression prediction model [8, 9]. Given the noisy and condition-specific nature of gene expression datasets, variants with small effects on gene expression may be undetectable at the sample sizes available. Additionally, the difference in sample size among transcriptome databases decreases our power to validate or discover more genes.

However, despite technical and population differences, most cis-eQTLs appear to be consistent between studies [66]. Therefore, despite in some cases a modest value of overall coefficient of correlation between predicted and measured gene expression, associations of the genetic component of gene expression as inferred by imputed gene expression have been successfully in identifying disease-associated genes that complement existing methods.

## Conclusions

In conclusion, we found that genetic determinants of gene expression were associated with severe COPD and quantitative emphysema phenotypes, identifying genes at known loci, and identifying novel COPD-associated genes. These findings were obtained by integrating GWAS results with gene expression data, performing colocalization analysis, and validating key results in independent gene expression datasets. These findings may provide mechanistic insights into the genetics of COPD.

## Additional file

Additional file 1: Supplementary Methods. **Table S1.** Colocalization probability and regulatory annotations for colocalized variants using corresponding GWAS and Lung-eQTL consortium datasets (see Excel file). **Table S2.** Colocalization probability and regulatory annotations for colocalized variants using corresponding GWAS and GTEx eQTL (blood and lung) datasets (see Excel file). **Table S3.** Additional imputed gene expression association results at previously described GWAS significant loci. **Table S4.** Result of association analysis between imputed gene expression and moderate to severe COPD of reported genes from severe COPD. **Table S5.** Differential expression analysis between COPD and controls using blood RNA-seq in COPDGene. **Table S6.** Differential expression analysis of quantitative emphysema using blood RNA-seq in COPDGene. **Table S7.** Differential expression analysis between severe COPD and controls using lung tissues. **Table S8.** Differential expression analysis of %LAA-950 and Perc15 using lung tissues. **Table S9.** T-cell-associated gene sets from Reactome using a ranked gene list from associations of %LAA-950 (DGN-Blood). **Table S10.** Covariate adjustments for differential expression analysis. **Figure S1.** Regional association plots within 50kb of *LILRA3*. GWAS of %LAA-950 and blood eQTL were shown in upper panel. Chromatin states and epigenomic marks of normal human lung fibroblast were shown in lower panel (see Supplementary Methods). **Figure S2.** Regional association plots within 50kb of *DCBLD1*. GWAS of %LAA-950 and lung eQTL were shown in upper panel. Chromatin states and epigenomic marks of normal human lung fibroblast were shown in lower panel (see Supplementary Methods). **Figure S3.** Scatter plot of effect size of significant SNPs from eQTL studies of blood and lung tissue for *FAM13A*. **Figure S4.** Contribution of each *FAM13A* SNP in prediction models to overall association statistics. (ZIP 758 kb)

## Abbreviations

%LAA-950: The percentage low attenuation area at − 950 HU threshold
CLPP: Colocalization posterior probability
COPD: Chronic obstructive pulmonary disease
COPDGene: Genetic Epidemiology of COPD
DGN-Blood: Whole blood from Depression Genes and Networks
eCAVIAR: eQTL and GWAS Causal Variant Identification in Associated Regions
ECLIPSE: Evaluation of COPD Longitudinally to Identify Predictive Surrogate Endpoints
eQTL: Expression quantitative trait loci
ER: Endoplasmic reticulum
GenKOLS: Genetics of COPD, Norway
GTEx-Blood: Whole blood from Genotype-Tissue Expression consortium
GTEx-Lung: Lung tissue from Genotype-Tissue Expression consortium
GWAS: Genome-wide association studies
HLA: Human Leukocyte Antigen
HU: Hounsfield units
LD: Linkage disequilibrium
MAPK: Mitogen-activated protein kinase
MHC: Major histocompatibility complex
NAS: Normative Aging Study
NETT: National Emphysema Treatment Trial
Perc15: The HU at the 15th percentile of the density histogram
TSS: Transcription start site
UPR: Unfolded protein response

## Footnotes

1 A preliminary abstract of this study was previously published: Sakornsakolpat P, Morrow JD, Castaldi PJ, Hersh CP, Silverman EK, Manichaikul A, et al. Integrative Analysis of Genomics and Transcriptomics Identifies Association of *PSMA4* with Emphysema. American Journal of Respiratory and Critical Care Medicine 2017;195:A7614. Available from: http://www.atsjournals.org.
