# Supplementary material for: Integrative genomics identifies new genes associated with severe COPD and emphysema
Source: Respir Res. 2018 Mar 22;19:46. doi: 10.1186/s12931-018-0744-9 (PMC5863845; doi:10.1186/s12931-018-0744-9)
Supplement: Supplementary file 1 — Supplementary Methods. Table S1. Colocalization probability and regulatory annotations for colocalized variants using corresponding GWAS and Lung-eQTL consortium datasets (see Excel file). Table S2. Colocalization probability and regulatory annotations for colocalized variants using corresponding GWAS and GTEx eQTL (blood and lung) datasets (see Excel file). Table S3. Additional imputed gene expression association results at previously described GWAS significant loci. Table S4. Result of association analysis between imputed gene expression and moderate to severe COPD of reported genes from severe COPD. Table S5. Differential expression analysis between COPD and controls using blood RNA-seq in COPDGene. Table S6. Differential expression analysis of quantitative emphysema using blood RNA-seq in COPDGene. Table S7. Differential expression analysis between severe COPD and controls using lung tissues. Table S8. Differential expression analysis of %LAA-950 and Perc15 using lung tissues. Table S9. T-cell-associated gene sets from Reactome using a ranked gene list from associations of %LAA-950 (DGN-Blood). Table S10. Covariate adjustments for differential expression analysis. Figure S1. Regional association plots within 50kb of LILRA3. GWAS of %LAA-950 and blood eQTL were shown in upper panel. Chromatin states and epigenomic marks of normal human lung fibroblast were shown in lower panel (see Supplementary Methods). Figure S2. Regional association plots within 50kb of DCBLD1. GWAS of %LAA-950 and lung eQTL were shown in upper panel. Chromatin states and epigenomic marks of normal human lung fibroblast were shown in lower panel (see Supplementary Methods). Figure S3. Scatter plot of effect size of significant SNPs from eQTL studies of blood and lung tissue for FAM13A. Figure S4. Contribution of each FAM13A SNP in prediction models to overall association statistics. (ZIP 758 kb) [file 12931_2018_744_MOESM1_ESM.zip › Sakornsakolpat.PrediXcanSupplement.doc]

# Supplementary Methods

## Genome-wide association studies and meta-analysis

Genome-wide associations studies were as previously described [1,2]. In total, we included 3,497 individuals with severe COPD and 5,704 controls, and 12,031 individuals for the analysis of quantitative emphysema (combined cases and non-cases). We also included 6,633 individuals with moderate to severe COPD and 5,704 control individuals to assess the associations in less severe COPD [1].

## Integration of GWAS and gene expression

We used S-PrediXcan to integrate GWAS and gene expression data. Briefly, we used the z-scores from GWAS summary statistics and combined them with their corresponding contributing weights. Weights determine the contribution of each variant to imputed gene expression calculated using Elastic Net based on *cis-* variants (i.e., located +/-1 Mb around a gene) as the predictors and corresponding gene expression level as the outcome. The relationship between regression coefficients from input GWAS, weights from Elastic Net model, and resulting z-scores in S-PrediXcan (as described in [3]) is:

while is the weight of SNP from the prediction model of gene, is the regression coefficients for SNP from GWAS, is the standard error of, is the estimated variance of SNP, is the estimated variance of the predicted expression of gene.

While lung tissue is the most relevant for COPD and emphysema, several studies have identified COPD-related differences in peripheral blood [4,5] and blood datasets have larger sample sizes, which results in a greater number of significant eQTLs and more accurate gene / transcriptome predictions [6–8]. Given the fact that power increases with sample size and large scale COPD-specific blood expression dataset is underway [9], we utilized the largest blood expression database that publicly available, DGN-Blood. This database was built on whole blood RNA-seq data of 922 individuals diagnosed with major depressive disorder [10]. GTEx-Lung is based on multi-tissue RNA-seq studies from the GTEx consortium (V6p release)[6] on 278 lung tissue samples. For our discovery analysis, prediction models in DGN-Blood and GTEx-Lung were trained on single nucleotide polymorphisms (SNPs) from HapMap 3 [11] and located within 1 Mb upstream and downstream of each gene. The training of prediction models was done by glmnet package in R with mixing parameter alpha = 0.5, and the 10-fold cross-validation was used in choosing the penalty parameter lambda. Once all genes were trained, we retained only genes with significant prediction (using predicted gene expression as the predictor and measured gene expression as the outcome) with false discovery rate less than 0.05.

## Validation in other reference transcriptome databases

To validate the significant associations from discovery stage, we further used independent datasets, GTEx for whole blood (GTEx-Blood) and the Lung-eQTL Consortium for lung tissue. GTEx-Blood contains 338 whole blood samples; methods for generating weights are as described above. The Lung-eQTL consortium is a collaborative effort from Laval University, University of British Columbia, and University of Groningen [12] and contains 1,111 lung tissue samples. Lung eQTL consortium weights were generated using the Haplotype Reference Consortium (HRC) reference panel version 1.1.[8] and gene association results were generated using TWAS/FUSION [13] as these methods are similar. We used the parameters for model training as described in the previous section.

## Colocalization analysis using eCAVIAR

We used eCAVIAR (eQTL and GWAS Causal Variant Identification in Associated Regions) for the colocalization analysis (Methods). We used the recommended parameters stated in the paper [14] and the software’s documentation (downloaded on December 23, 2016). For the colocalization posterior probability (CLPP), we used 0.01 or greater, as this has been demonstrated to have high accuracy and precision (19). To maximize computational efficiency, we selected GWAS variants with absolute z-scores greater than 4 and set the maximal number of causal variants to four. We approximated the LD structure in our GWAS and eQTL analysis using European samples from the 1000 Genomes Project Phase 3v5.

## Variant functional annotation

We searched the HaploReg database for an overlap with promotor/enhancer histone marks from NIH Roadmap Epigenomics Mapping Consortium (ROADMAP) and Encyclopedia of DNA Elements Consortium (ENCODE), DNAse hypersensitivity sites, protein-protein interactions, motifs interruption, NHGRI/EBI GWAS hits, GRASP QTL hits, and publicly available eQTL hits curated in HaploReg 4.1 [15]. Regional association plots of GWAS and eQTL datasets with epigenomic annotations were created using Enlight [16]. As shown in **Table S1** and **S2**, colocalized variants overlapped with various lung relevant cell types. For illustration, we presented regulatory marks of normal human lung fibroblasts in the figures. We showed DNase-seq (accessible regions), H3k4me3 (promoters), H3k4me1 (enhancers), CTCF (bind to insulators), H3k27me3 (repressors), and H3k27ac (enhancers) from ChIP-seq.

## Pathway enrichment analysis of S-PrediXcan results

To determine whether phenotype-associated genetic variants perturbed specific sets of genes, we analyzed the top 1% of differentially expressed genes for each phenotype and reference transcriptome database. We used gProfileR [17] to perform pathway enrichment analysis by an overrepresentation method using the functional categories from Gene Ontology (GO), Reactome database, and Kyoto Encyclopedia of Genes and Genomes (KEGG) pathway. We used a g:SCs (Set Counts and Sizes) threshold at 0.05 as it performs better than other metrics (e.g., FDR) in determining the significance of enrichment [17]. We also assessed whether the associated gene set (from the analysis by gProfileR) is up- or down-regulated using Gene Set Enrichment Analysis or GSEA [18]. We obtained the rank of genes from S-PrediXcan results while considering the magnitude and signs of Z-score. The default parameters were used.

## Differential expression analysis of significant associations

We also sought to investigate the associations of gene expression and COPD/quantitative emphysema in significant associations from the S-PrediXcan analysis. We performed differential expression analysis using blood RNA-seq in COPDGene [9] and lung tissues [19]. Details on technical procedures, quality control, and statistical analysis were published earlier [9,19]. Sample size and covariate adjustments for each phenotype tested in this analysis were summarized in **Table S10**.

## Opposite direction of eQTLs in blood and lung tissue

To confirm the opposite direction of effects of genetically regulated *FAM13A* expression, we leveraged two large eQTL studies of blood and lung tissue: Framingham Heart Study (FHS) [20] and Lung-eQTL Consortium [12]. We extracted significant SNPs that are present in both studies, and plotted their effect sizes (blood in x axis and lung tissue in y axis). We also curated the SNP-level association statistics of S-PrediXcan/TWAS results to assess the contribution of each SNP to overall genetic component of gene expression (predicted gene expression).

# References

1. Cho MH, McDonald M-LLN, Zhou X, Mattheisen M, Castaldi PJ, Hersh CP, et al. Risk loci for chronic obstructive pulmonary disease: a genome-wide association study and meta-analysis. Lancet Respir Med [Internet]. 2014;2:214–25. Available from: https://www.ncbi.nlm.nih.gov/pubmed/24621683

2. Cho MH, Castaldi PJ, Hersh CP, Hobbs BD, Barr RG, Tal-Singer R, et al. A Genome-Wide Association Study of Emphysema and Airway Quantitative Imaging Phenotypes. Am. J. Respir. Crit. Care Med. [Internet]. 2015;192:559–69. Available from: http://www.ncbi.nlm.nih.gov/pubmed/26030696

3. Barbeira A, Dickinson SP, Torres JM, Torstenson ES, Zheng J, Wheeler HE, et al. Integrating tissue specific mechanisms into GWAS summary results. bioRxiv. 2017;

4. Castaldi PJ, Cho MH, Zhou X, Qiu W, McGeachie M, Celli B, et al. Genetic control of gene expression at novel and established chronic obstructive pulmonary disease loci. Hum Mol Genet [Internet]. 2015;24:1200–10. Available from: https://www.ncbi.nlm.nih.gov/pubmed/25315895

5. Bahr TM, Hughes GJ, Armstrong M, Reisdorph R, Coldren CD, Edwards MG, et al. Peripheral blood mononuclear cell gene expression in chronic obstructive pulmonary disease. Am. J. Respir. Cell Mol. Biol. [Internet]. 2013;49:316–23. Available from: http://www.ncbi.nlm.nih.gov/pubmed/23590301

6. Melé M, Ferreira PG, Reverter F, DeLuca DS, Monlong J, Sammeth M, et al. Human genomics. The human transcriptome across tissues and individuals. Science [Internet]. 2015;348:660–5. Available from: http://www.ncbi.nlm.nih.gov/pubmed/25954002

7. Gamazon ER, Wheeler HE, Shah KP, Mozaffari S V, Aquino-Michaels K, Carroll RJ, et al. A gene-based association method for mapping traits using reference transcriptome data. Nat Genet [Internet]. 2015;47:1091–8. Available from: https://www.ncbi.nlm.nih.gov/pubmed/26258848

8. Gusev A, Ko A, Shi H, Bhatia G, Chung W, Penninx BWJH, et al. Integrative approaches for large-scale transcriptome-wide association studies. Nat. Genet. [Internet]. 2016;48:245–52. Available from: http://www.ncbi.nlm.nih.gov/pubmed/26854917

9. Parker MM, Chase RP, Lamb A, Reyes A, Saferali A, Yun JH, et al. RNA sequencing identifies novel non-coding RNA and exon-specific effects associated with cigarette smoking. BMC Med. Genomics [Internet]. 2017;10:58. Available from: http://www.ncbi.nlm.nih.gov/pubmed/28985737

10. Battle A, Mostafavi S, Zhu X, Potash JB, Weissman MM, McCormick C, et al. Characterizing the genetic basis of transcriptome diversity through RNA-sequencing of 922 individuals. Genome Res [Internet]. 2014;24:14–24. Available from: https://www.ncbi.nlm.nih.gov/pubmed/24092820

11. International HapMap 3 Consortium, Altshuler DM, Gibbs RA, Peltonen L, Altshuler DM, Gibbs RA, et al. Integrating common and rare genetic variation in diverse human populations. Nature [Internet]. 2010;467:52–8. Available from: http://www.ncbi.nlm.nih.gov/pubmed/20811451

12. Hao K, Bossé Y, Nickle DC, Paré PD, Postma DS, Laviolette M, et al. Lung eQTLs to help reveal the molecular underpinnings of asthma. PLoS Genet. [Internet]. 2012;8:e1003029. Available from: http://www.ncbi.nlm.nih.gov/pubmed/23209423

13. Obeidat M, Lamontagne M, Bérubé J-C, Cho MH, Hobbs BD, de Jong K, et al. Leveraging lung tissue transcriptome to uncover candidate causal genes in COPD genetic associations. bioRxiv [Internet]. 2017; Available from: http://biorxiv.org/content/early/2017/09/26/193938.abstract

14. Hormozdiari F, van de Bunt M, Segrè A V, Li X, Joo JWJ, Bilow M, et al. Colocalization of GWAS and eQTL Signals Detects Target Genes. Am. J. Hum. Genet. [Internet]. 2016;99:1245–60. Available from: http://www.ncbi.nlm.nih.gov/pubmed/27866706

15. Ward LD, Kellis M. HaploReg: a resource for exploring chromatin states, conservation, and regulatory motif alterations within sets of genetically linked variants. Nucleic Acids Res. [Internet]. 2012;40:D930-4. Available from: http://www.ncbi.nlm.nih.gov/pubmed/22064851

16. Guo Y, Conti D V, Wang K. Enlight: web-based integration of GWAS results with biological annotations. Bioinformatics [Internet]. 2015;31:275–6. Available from: http://www.ncbi.nlm.nih.gov/pubmed/25262152

17. Reimand J, Arak T, Adler P, Kolberg L, Reisberg S, Peterson H, et al. g:Profiler-a web server for functional interpretation of gene lists (2016 update). Nucleic Acids Res. [Internet]. 2016;44:W83-9. Available from: http://www.ncbi.nlm.nih.gov/pubmed/27098042

18. Subramanian A, Tamayo P, Mootha VK, Mukherjee S, Ebert BL, Gillette MA, et al. Gene set enrichment analysis: a knowledge-based approach for interpreting genome-wide expression profiles. Proc. Natl. Acad. Sci. U. S. A. [Internet]. 2005;102:15545–50. Available from: http://www.ncbi.nlm.nih.gov/pubmed/16199517

19. Morrow JD, Zhou X, Lao T, Jiang Z, DeMeo DL, Cho MH, et al. Functional interactors of three genome-wide association study genes are differentially expressed in severe chronic obstructive pulmonary disease lung tissue. Sci Rep [Internet]. 2017;7:44232. Available from: https://www.ncbi.nlm.nih.gov/pubmed/28287180

20. Joehanes R, Zhang X, Huan T, Yao C, Ying S-X, Nguyen QT, et al. Integrated genome-wide analysis of expression quantitative trait loci aids interpretation of genomic association studies. Genome Biol. 2017;18:16.

**Supplementary Tables and Figures**

**Table S1 Colocalization probability and regulatory annotations for colocalized variants using corresponding GWAS and Lung-eQTL consortium datasets (see Excel file)**

**Table S2 Colocalization probability and regulatory annotations for colocalized variants using corresponding GWAS and GTEx eQTL (blood and lung) datasets (see Excel file)**

**Table S3 Additional imputed gene expression association results at previously described GWAS significant** loci

| **Phenotype** | **Genetic Loci** | **Gene** | **Tissue** | **Z score** | **P value** |
| --- | --- | --- | --- | --- | --- |
| **Severe COPD** | 1q41 | *TGFB2* | Lung | -5.2 | 2.00E-07 |
| **Severe COPD** | 4q31 | *ANAPC10* | Blood | -2.78 | 5.40E-03 |
| **Severe COPD** | 4q31 | *GYPA* | Blood | -4.57 | 4.80E-06 |
| **Severe COPD** | 4q31 | *HHIP* | Lung | 5.98 | 2.30E-09 |
| **Severe COPD** | 14q32 | *RIN3* | Blood | 2.39 | 1.70E-02 |
| **%LAA-950** | 4q31 | *GYPA* | Blood | -3.05 | 2.30E-03 |
| **%LAA-950** | 4q31 | *HHIP* | Lung | 5.24 | 1.60E-07 |
| **Perc15** | 4q31 | *GYPA* | Blood | 2.35 | 1.90E-02 |
| **Perc15** | 4q31 | *HHIP* | Lung | -4.9 | 9.40E-07 |

Blood and Lung correspond to reference transcriptome databases from DGN-Blood and Lung-eQTL Consortium, respectively.

**Table S4** Result of association analysis between imputed gene expression and moderate to severe COPD of reported genes from severe COPD

| **Tissue** | **Gene** | **Moderate to severe COPD** | | **Severe COPD** | |
| --- | --- | --- | --- | --- | --- |
|  |  | Effect Size | Effect Size | Effect Size | P Value |
| **Blood** | *HYKK* | -1.355 | 1.97E-13 | -1.887 | 8.16E-17 |
| **Blood** | *EGLN2* | 0.259 | 2.86E-05 | 0.367 | 1.03E-06 |
| **Blood** | *FAM13A* | -0.557 | 3.18E-08 | -0.681 | 4.81E-08 |
| **Blood** | *PSMA4* | 0.645 | 3.78E-10 | 0.971 | 2.47E-14 |
| **Blood** | *RAB4B* | -0.561 | 1.50E-05 | -0.756 | 1.72E-06 |
| **Lung** | *GPRIN3* | 3.983 | 5.28E-06 | 5.381 | 7.43E-07 |
| **Lung** | *WNT3* | 0.321 | 7.29E-05 | 0.488 | 1.24E-06 |

**Table S5 Differential expression analysis between COPD and controls using blood RNA-seq in COPDGene. Nominal significant associations are in bold; FC = fold change of expression values; FDR = false discovery rate.**

| **Gene** | **logFC** | **P Value** | **FDR** |
| --- | --- | --- | --- |
| ***HYKK*** | 0.043 | 0.548 | 0.953 |
| ***PSMA4*** | 0.025 | 0.133 | 0.817 |
| ***FAM13A*** | -0.02 | 0.492 | 0.948 |
| ***GPRIN3*** | 0.018 | 0.332 | 0.915 |
| ***EGLN2*** | -0.002 | 0.899 | 0.992 |
| ***WNT3*** | -0.084 | 0.205 | 0.863 |
| ***RAB4B*** | -0.024 | 0.326 | 0.914 |

**Table S6 Differential expression analysis of quantitative emphysema using blood RNA-seq in COPDGene Nominal significant associations are in bold; FC = fold change of expression values; FDR = false discovery rate.**

| **Phenotype** | **Gene** | **logFC** | **P Value** | **FDR** |
| --- | --- | --- | --- | --- |
| **%LAA-950** | *HYKK* | -0.005 | 0.807 | 0.986 |
| **%LAA-950** | *PSMA4* | -0.012 | 0.028 | 0.662 |
| **%LAA-950** | *ITGA1* | -0.023 | 0.211 | 0.875 |
| **%LAA-950** | *ATF6B* | -0.001 | 0.889 | 0.995 |
| **%LAA-950** | *DCBLD1* | 0.012 | 0.377 | 0.922 |
| **Perc15** | *HYKK* | -0.002 | 0.279 | 0.902 |
| **Perc15** | *PSMA4* | 0.001 | 0.082 | 0.786 |
| **Perc15** | *ITGA1* | 0.001 | 0.298 | 0.906 |
| **Perc15** | *ATF6B* | 0 | 0.994 | 1 |
| **Perc15** | *DCBLD1* | -0.001 | 0.309 | 0.909 |

**Table S7 Differential expression analysis between severe COPD and controls using lung tissues. Nominal significant associations are in bold; FC = fold change of expression values; FDR = false discovery rate.**

| **Gene** | **Probe ID** | **logFC** | **P Value** | **FDR** |
| --- | --- | --- | --- | --- |
| ***HYKK*** | ILMN_2091707 | -0.266 | 0.075 | 0.513 |
| ***HYKK*** | ILMN_1708351 | 0.161 | 0.137 | 0.625 |
| ***PSMA4*** | ILMN_1682098 | -0.027 | 0.663 | 0.935 |
| ***PSMA4*** | ILMN_2198376 | 0.018 | 0.763 | 0.961 |
| ***FAM13A*** | ILMN_1800267 | -0.087 | 0.336 | 0.8 |
| ***FAM13A*** | ILMN_2401258 | 0.133 | 0.354 | 0.809 |
| ***FAM13A*** | ILMN_2401253 | -0.062 | 0.692 | 0.942 |
| ***FAM13A*** | ILMN_1752510 | 0.054 | 0.694 | 0.943 |
| ***GPRIN3*** | ILMN_1776435 | 0.356 | 0.12 | 0.6 |
| ***RAB4B-EGLN2*** | ILMN_2354391 | -0.051 | 0.424 | 0.843 |
| ***RAB4B-EGLN2*** | ILMN_1768773 | -0.035 | 0.662 | 0.935 |
| ***WNT3*** | ILMN_1803593 | -0.112 | 0.307 | 0.783 |

**Table S8 Differential expression analysis of %LAA-950 and Perc15 using lung tissues.** Nominal significant associations are in bold; FC = fold change of expression values; FDR = false discovery rate.

| **Phenotype** | **Gene** | **Probe ID** | **logFC** | **P Value** | **FDR** |
| --- | --- | --- | --- | --- | --- |
| **%LAA-950** | *HYKK* | ILMN_2091707 | -0.971 | 0.078 | 0.404 |
| **%LAA-950** | *HYKK* | ILMN_1708351 | -0.451 | 0.186 | 0.564 |
| **%LAA-950** | *PSMA4* | ILMN_1682098 | -0.278 | 0.194 | 0.574 |
| **%LAA-950** | *PSMA4* | ILMN_2198376 | -0.133 | 0.526 | 0.816 |
| **%LAA-950** | *ATF6B* | ILMN_1814313 | -0.586 | 0.132 | 0.495 |
| **%LAA-950** | *ATF6B* | ILMN_3273641 | 0.268 | 0.404 | 0.746 |
| **%LAA-950** | *ATF6B* | ILMN_3194911 | 0.213 | 0.487 | 0.797 |
| **%LAA-950** | *DCBLD1* | ILMN_1891927 | -0.217 | 0.317 | 0.685 |
| **%LAA-950** | *DCBLD1* | ILMN_1695604 | -0.097 | 0.855 | 0.957 |
| **%LAA-950** | ***LILRA3*** | **ILMN_1786303** | **-1.956** | **0.034** | **0.298** |
| **%LAA-950** | *LILRA3* | ILMN_3237878 | -1.569 | 0.105 | 0.453 |
| **%LAA-950** | *LILRA3* | ILMN_1661631 | -2.041 | 0.108 | 0.46 |
| **Perc15** | *HYKK* | ILMN_2091707 | 0.004 | 0.159 | 0.561 |
| **Perc15** | *HYKK* | ILMN_1708351 | 0.001 | 0.395 | 0.759 |
| **Perc15** | *PSMA4* | ILMN_1682098 | 0.001 | 0.155 | 0.557 |
| **Perc15** | *PSMA4* | ILMN_2198376 | 0 | 0.664 | 0.895 |
| **Perc15** | *ATF6B* | ILMN_1814313 | 0.002 | 0.275 | 0.677 |
| **Perc15** | *ATF6B* | ILMN_3194911 | -0.001 | 0.309 | 0.706 |
| **Perc15** | *ATF6B* | ILMN_3273641 | 0 | 0.905 | 0.974 |
| **Perc15** | *DCBLD1* | ILMN_1891927 | 0.001 | 0.255 | 0.661 |
| **Perc15** | *DCBLD1* | ILMN_1695604 | -0.002 | 0.532 | 0.839 |
| **Perc15** | *LILRA3* | ILMN_1786303 | 0.008 | 0.057 | 0.392 |
| **Perc15** | *LILRA3* | ILMN_1661631 | 0.009 | 0.13 | 0.525 |
| **Perc15** | *LILRA3* | ILMN_3237878 | 0.006 | 0.17 | 0.573 |

**Table S9** T-cell-associated gene sets from Reactome using a ranked gene list from associations of %LAA-950 (DGN-Blood)

| **Pathway** | **Size** | **Enrichment Score** | **Normalized Enrichment Score** | **Nominal P Value** | **FDR** | **Rank at max** |
| --- | --- | --- | --- | --- | --- | --- |
| **TCR signaling** | 40 | 0.355 | 1.372 | 0.062 | 1 | 1054 |
| **MHC class II antigen presentation** | 69 | 0.320 | 1.331 | 0.066 | 1 | 1639 |
| **Downstream TCR signaling** | 27 | 0.369 | 1.259 | 0.138 | 1 | 1054 |

**Table S10 Covariate adjustments** for differential expression analysis

| Tissue | Phenotype | Number of samples | Covariates |
| --- | --- | --- | --- |
| Blood [9] | Moderate-to-severe COPD (GOLD 2-4) | 238 cases and 226 controls | Age, gender, race, pack-years of smoking history, current smoking status, total white blood cell count, lymphocyte percentage, monocyte percentage, neutrophil percentage, eosinophil percentage, library construction batch, and significant surrogate variables |
|  | %LAA-950 | 486 individuals | Age, gender, race, current smoking status, body mass index, computed tomography scanner model, library construction batch, and significant surrogate variables |
|  | Perc15 | 486 individuals | Age, gender, race, current smoking status, body mass index, computed tomography scanner model, library construction batch, and significant surrogate variables |
| Lung [19] | Severe COPD (GOLD 3-4) | 111 cases and 40 controls | Age, gender, race, pack-years of smoking history, and significant surrogate variables |
|  | %LAA-950 | 151 individuals | Age, gender, race, pack-years of smoking history, body mass index, and significant surrogate variables |
|  | Perc15 | 151 individuals | Age, gender, race, pack-years of smoking history, body mass index, and significant surrogate variables |

**Figure S1 Regional association plots within 50kb of *LILRA3*. GWAS of %LAA-950 and blood eQTL were shown in upper panel. Chromatin states and epigenomic marks of normal human lung fibroblast were shown in lower panel (see Supplementary Methods).**

**Figure S2 Regional association plots within 50kb of *DCBLD1*. GWAS of %LAA-950 and lung eQTL were shown in upper panel. Chromatin states and epigenomic marks of normal human lung fibroblast were shown in lower panel (see Supplementary Methods).**

**Figure S3 Scatter plot of effect size of significant SNPs from eQTL studies of blood and lung tissue for *FAM13A*.** Blue dots are SNPs that were used in prediction models of gene expression in blood or lung tissue. Most SNPs have opposite direction of effect size in blood and lung tissue except two SNPs (rs10016030 and rs998654 which contributed minimally to the overall association Z-score – see Figure S4). This finding supports the opposite direction of effect identified using S-PrediXcan and TWAS.

**Figure S4 Contribution of each *FAM13A* SNP in prediction models to overall association statistics**. The upper panel shows SNPs in the prediction model of lung tissue and blood in the lower panel. The direction of effect (sign of association Z-score) is expressed as the red (up-regulation) and blue (down-regulation). The size of the SNP corresponds to assigned weight in the prediction model. SNPs with major contribution have high absolute Z-score (y-axis) with high GWAS z-score or high weight (or both). Note the SNPs with concordant direction of effect in both tissues (rs10016030 in lung tissue and rs998654 in blood) contributed minimally to the association Z-score.
